# Profiles of well-being in French older adults and associations with successful aging and personality: findings from the SHARE project

**DOI:** 10.1007/s40520-024-02705-x

**Published:** 2024-03-29

**Authors:** Elina Van Dendaele, Kristell Pothier, Nathalie Bailly

**Affiliations:** PAVeA Laboratory, EA 2114, Department of Psychology, University of Tours, 3, Rue Des Tanneurs BP 4103, 37041 Tours Cedex 1, France

**Keywords:** Well-being, Successful aging, Personality, Older adults

## Abstract

Maintaining the well-being of the older adults is a primary concern in gerontology. This study determined different profiles of well-being (WB) and compared the profiles in terms of successful aging (SA), personality, and sociodemographic variables. The study sample consisted of 856 adults aged 65–98 years. WB was taken into account in an eudemonic and hedonic approach. SA was measured by assessing the three distinct components of Rowe and Kahn's model (Successful aging. Gerontol 37(4):433–440. 10.1093/geront/37.4.433, 1997), personality by the Big Five Inventory, and sociodemographic variables. Latent class analyses (LCA) determined the number of WB profiles, and ANOVAs and Chi2 tests to compare them. The LCA revealed three WB profiles: Profile 1 (9.35%, n = 80), Profile 2 (37.38%, n = 320), and Profile 3 (53.27%, n = 456) in which participants reported lower, intermediate, and higher WB scores. Our results confirm that a high level of WB (Profile 3) can be linked to the components of SA and socio-demographic characteristics (age, marital status, level of education, income). This raises questions about the injunctions concerning healthy aging that older people integrate. It's also interesting to note that the intermediate profile (profile 2) can be either close to the "lower WB" profile (Profile 1) in terms of openness, conscientiousness, and agreeableness or to the "higher WB" profile (Profile 3) in terms of extraversion. However the three profiles do not have the same level of neuroticism. These results also showed the importance of adapting the support offered to older people according to their health status and/or individual characteristics.

## Background

As life expectancy increases, it is important to maintain the well-being (WB) of aging people as long as possible. There are two approaches to WB: the eudemonic approach refers to the importance of WB and personal growth throughout life, with specific criteria like self-realization, or control to guarantee psychological WB (PWB). The hedonic approach corresponds to the level of pleasure, comfort and happiness experienced. It is made up of a cognitive aspect (life satisfaction) and an affective aspect (pleasure) [1], we speak of subjective WB (SWB). Given the intra- and inter-individual variability of older adults, each aging trajectory could lead to a different state of WB from one individual to another. In short, the PWB and SWB are conceptually linked, but differ in the type of experience they involve. It therefore seems necessary to consider both eudemonic and hedonic indicators of PWB to understand the multifaceted and multidirectional dynamics of PWB in very old age [2]. Indeed, the literature shows a heterogeneity of WB: When PWB is assessed, it is found to decline more during old age [3, 4]. On the other hand, studies show that over time, SWB does not decline overall [5–8]. It is thought to evolve in an inverted U-shape or linearly between individuals [9], and longitudinal studies examining changes in subjective well-being have concluded that age can predict increases in life satisfaction [10]. Finally, SWB differs between older people, meaning that variables play a role in this.

Since the 1950s, studies in gerontology have focused on the behaviors that individuals put in place to cope with aging and promote their well-being. Among these models, Rowe and Kahn's [11], describes "successful aging" (SA) as a state, an objective and measurable condition. Their model proposes three components for SA: a low probability of disease and disease-related disability, high cognitive and physical functional capacity, and active engagement in life. Although the absence of disease and disability associated with good health has been identified as a predictor of WB, this normative view of healthy aging allows few older adults to meet this definition [12]. Indeed, some studies have highlighted limitations regarding the components of SA, as the model does not include older adults’ own perceptions of SA [13].

In addition to these specific determinants of SA, other variables can also influence WB [14]. Personality is one of the most important and consistent predictors of WB [15]. This could be explained by the fact that certain personality traits promote health behaviors or mental and physical health [16], which in turn promote personal WB. Personality can be divided into five main traits when the Big Five are used to measure it [17]: openness (tendency to be imaginative, curious, and open to new ideas,conscientiousness (tendency to be organized, cautious, and diligent; extraversion (tendency to be sociable and assertive; agreeableness (tendency to be confident and cooperative; and neuroticism (tendency to experience negative emotional states such as nervousness, anxiety, depression, or insecurity. Personality may compensate for the losses experienced by some older people and explain various aspects of a healthy aging process [18–20]. A recent study showed that the main factors influencing WB (measured by life satisfaction and positive and negative affects) in older adults were mainly related to personality traits, with “neuroticism” being the variable with the greatest negative predictive power for WB, in particular because it is more difficult for people with high neurotic tendencies to perceive the positive aspects of their lives and they focus more on the negative aspects [21]. This could be explained by the fact that neurotic people have more physical symptoms, have a poorer perception of their health and complain more about health problems, are more preoccupied with their health and pay more attention to it, and have poorer physical health.

In addition, the WHO signifies the importance of considering intrinsic and extrinsic variables when addressing healthy aging [3] (2015). For these reasons, sociodemographic variables could also have an impact on WB in older adults. Indeed, living with a spouse/partner would have a positive influence on WB, notably due to the positive emotions it provides [22, 23]. Environmental characteristics are also important for WB as annual household income, education level, and living environment are significant predictors of WB as they may promote access to social support and social engagement.

## Aims

Until now, the above-mentioned factors influencing healthy ageing have not been studied together. The aim of the present study was therefore, in an integrated approach, to improve the understanding of WB in the context of aging. The present study had two objectives: (1) to identify different profiles based on both psychological and subjective components of WB; and (2) to compare WB profiles in terms of successful aging, personality, and sociodemographic variables.

## Methods

### Participants

Characteristics of the study population are described in Table 1. 856 French participants ([65–98]; mean = 74.99 years old, standard deviation (SD = 7.42), living independently in their own homes, were selected from the seventh wave of Survey of Health, Aging, and Retirement in Europe (SHARE). 56.54% were female (n = 484) and 43.46% were male (n = 372). The mean level of education (using the International Standard Classification of Education, ISCED) was 2.7 ([0–6]; SD = 1.78). Almost 69% (n = 538) of participants lived with their spouse or partner. The average household income was 34,624 euros ([0; 154,656] SD = 21,523.88) per annum.

**Table 1:** Characteristics of the study population

	Mean (SD) or % (n)
Age	74.99 (7.42)
Education level	2.79 (1.75)
Gender (female)	56.54% (n = 484)
Marital status (living with spouse/partner) (n = 782)	68.8% (n = 538)
Household income (in one year)	34,624.48 (21,523.88)
Psychological well-being	
CASP-12	
Control [1–12]	8.17 (2.33)
Self-realization [1–12]	9.44 (2.09)
Subjective well-being	
CASP-12	
Pleasure [1–12]	10.52 (1.74)
Life satisfaction [0–10]	7.41 (1.53)
Self-perceived health [0–5]	2.75 (0.97)
Cognitive function	
Verbal fluency [number of words 0–100]	18.54 (7.2)
Numeracy score	4.03 (1.37)
Learning a list of 10 words	5.16 (1.69)
Delayed recall of a list of 10 words—total	3.74 (2.08)
Physical function	
Chronic illness [number of diseases]	2.04 (1.51)
Grip strength (kilograms)	29.66 (11.27)
Activity limitation [Strongly limited %]	50.58% (n = 433)
Engagement with life [0–1]	
Social activities [Yes %]	86.21% (n = 738)
Support given [Yes %]	32.59% (n = 279)
Support received [Yes %]	29.09% (n = 249)
Personality traits [0–5]	
Openness	3.59 (0.94)
Conscientiousness	4.43 (0.71)
Extraversion	3.46 (0.863)
Agreeableness	3.9 (0.8)
Neuroticism	3.03 (1.09)

*Education level* SHARE uses the International Standard Classification of Education (ISCED), which allows standardized reporting of education statistics according to an internationally agreed set of definitions and concepts, *ISCED 1* Primary education, *ISCED 2* Lower secondary education, *ISCED 3* Upper secondary education, *ISCED 4* Post-secondary non-tertiary education, *ISCED 5* Short-cycle tertiary education, *ISCED 6* Bachelor’s or equivalent level, *ISCED 7* Master’s or equivalent level, *ISCED 8* Doctoral or equivalent level

### Measures

*Well-Being* was measured using three distinct measures:

- Control, Self-realization, and Pleasure were measured with the scale CASP-12 version suggested by Wiggins et al. [24, 25]. Each item is answered on a four-point Likert scale (1 = never to 4 = often). In the current study, the CASP-12 showed acceptable to good psychometric properties, as Cronbach α values were 0.68 for control, 0.68 for pleasure, 0.76 for self-realization.
- Life Satisfaction was measured with one item “Can you rate your quality of life, on a scale from 0 to 10, (where 0 means you are completely dissatisfied and 10 means you are completely satisfied with your life)?”. Higher life satisfaction values indicate a better life satisfaction.
- Self-perceived health was measured with the question: “How is your health in general?”, using a 5-point Likert scale (1 = *very bad* to 5 = *very good*.). Higher self-perceived health values indicate a better perceived health.

Single-item measures of subjective well-being are widely used in the literature (Cooper et al., 2011) and show very similar performance compared to multiple items [26, 27].

*Successful Aging *was measured by evaluating the three distinct components of Rowe and Kahn’s model [11]:

- *Disease* was measured through the number of chronic illnesses, self-reported using the standardized question “Have you ever been diagnosed with one of the following conditions?” with the following answers: 1. heart disease, coronary insufficiency, angina pectoris or myocardial infarction or any other heart problem, including heart failure 2. Hypertension, high blood pressure 3. Cholesterol, hypercholesterolemia 4. Stroke or cerebrovascular disease, stroke 5. Diabetes, high blood sugar 6. chronic lung disease, such as chronic bronchitis or emphysema 7. Asthma 8. Arthritis, including osteoarthritis and rheumatism 9. Osteoporosis 10. Cancer or malignant tumor, including leukemia or lymphoma, excluding benign skin cancers 11. Gastric or duodenal ulcer 12. Parkinson's disease 13. Cataract 14. Hip fracture
- *Cognitive capacity* was measured with four indicators evaluating executive and attentional functions, and memory. Executive and attentional functions were assessed by (1) a verbal fluency test, in which participants had to name as many animals as possible in 60 s [0–100]; and (2) arithmetic calculations. Respondents were asked to perform four calculations [28]. Based on responses to these four questions, a numeracy score ranging from one to five (5 = good skills) was constructed. Memory was assessed by an immediate and delayed (after completion of the two additional cognitive tasks) recall test of 10 words.
- *Physical functional capacity* was measured using two indicators (1) long-term activity limitations related to health status assessed through the Global Activity Limitation Indicator (GALI, [29] with the following question: “For the past 6 months or more, have you been limited your everyday activities because of a health problem?” [1: Yes, strongly limited/limited, 0: No, not limited]. (2) Grip strength was measured using a Smedley handheld dynamometer (100 kg). Participants were asked to stand or sit with their arms clasped against their trunk, elbows at a 90° angle, and then to squeeze the handles of the dynamometer as strongly as possible for five seconds. This was repeated four times and the maximum score was retained [19].
- *Engagement with life* was measured with three indicators: (1) Social activities were measured through four questions regarding volunteering, sports, politics, and religion [0: no social activity; 1: at least one social activity]. (2) Given and (3) received support were measured and characterized according to the answers corresponding to the help given and/or received, in the previous 12 months, to/from a person, family member, friend, or neighbor, outside or inside the household [0: no / 1: yes]. This gave three percentages of the number of people who were considered to be "engaged in life".

*Personality* was assessed using the French version of the 10-item Big-Five Inventory (BFI-10; [30], a self-report measure that assesses openness, conscientiousness, extraversion, agreeableness and neuroticism (two questions per trait). The BFI-10 has demonstrated good reliability and validity across different samples (test–retest correlations ≥ 0.65 across scales [31, 32]. Due to the brevity of the measure and because it has been developed by prioritizing content breadth over internal consistency, no Cronbach alpha was calculated [33]. This instrument uses a Likert-type scale [1: disagree strongly to 5: agree strongly] with a higher value representing a stronger correspondence with the trait.

*Sociodemographic variables* were assessed through chronological age, gender (male/female), level of education (ISCED), marital status (living with spouse/partner) and annual household income.

### Statistical analysis

Descriptive statistics for all variables were calculated using SPSS 26.

MPlus 8 [34] was used for latent class analysis (LCA) to describe profiles based on assessments of PWB (control, self-actualization) and SWB (pleasure, life satisfaction, perceived health). LCA allows the identification of different subgroups within populations that share certain outward characteristics [35]. Several statistical criteria were included to retain the number of classes. First, information criteria such as the Bayesian Information Criterion (BIC), sample size-adjusted BIC (ABIC), and Akaike Information Criterion (AIC) were used with smaller values indicating better fit. Second, we considered entropy, a statistic that assesses accuracy, and ranges from 0 to 1 (perfect accuracy). Finally, the Lo-Mendell-Rubin test (LMR) and the Bootstrap likelihood ratio test (BLRT) were also used to compare the improvement between models of neighboring classes, with a statistically significant result being interpreted as an improvement in the fit due to the additional class.

Once the number of latent classes was determined, groups of participants based on the classes (profiles) were compared in terms of determinants of SA, personality and sociodemographic variables. For the quantitative variables (i.e., cognitive abilities, number of chronic illnesses, grip strength, personality, age, level of education and household income), one-way ANOVAs were followed by Tukey’s post hoc tests to compare groups. For the categorical variables (i.e., activity limitations, engagement with life, gender and marital status) Chi^2^ tests were used.

## Results

### Well-being profiles

LCAs from one to five classes were conducted. Table 2 shows all the statistical criteria considered to decide the number of classes to be retained. The model with four classes had the lowest information criteria and had statistically significant LMR and BLRT tests. In addition, the 4- and 5-class models were also meaningful in terms of conceptual interpretability, but the fit was no better than for the 3-class model. Moreover, the best entropy data were obtained with three classes. Descriptive labels for profiles were then: Profile 1 (9.35%, n = 80), Profile 2 (37.38%, n = 320) and Profile 3 (53.27%, n = 456) in which participants reported lower, intermediate and higher WB scores, respectively (Table 2).

**Table 2:** Results of the latent class analysis

Class	Akaike (AIC)	Bayesian (BIC)	ABIC	Entropy	p for LMR	p for BLRT	Class size and assignment probability
One	16,422.623	16,470.146	16,438.389				
Two	15,479.363	15,555.399	15,504.587	0.840	< 0.001	< 0.001	215 (25.11%) 641 (74.88%)
Three	15,203.028	15,307.578	15,237.712	0.808	0.017	< 0.001	320 (37.38%) 80 (9.35%) 456 (53.27%)
Four	15,105.128	15,238.192	15,149.272	0.784	0.04	< 0.001	36 (4%) 146 (17%) 287 (33.52%) 387 (45.21%)
Five	15,021.849	15,183.426	15,075.452	0.799	.71	< .001	128 (14%) 45 (5%) 287 (33.52%) 161 (18.88%) 391 (45.67%) 131 (14.3%)

*AIC* Akaike information criterion, *BIC* Bayesian information criterion, *ABIC* adjusted BIC, *LMR* Lo-Mendell-Rubin test, *BLRT* bootstrapped log-likelihood ratio test

### WB profile differences with independent variables

One-way ANOVAs and Chi^2^ tests were conducted on the three WB profiles to compare them in terms of determinants of SA, personality and sociodemographic variables (Table 3).

**Table 3:** Effect of well-being profile on mean scores for SA components, personality and sociodemographic variables

	Profile 1: Lower WB n = 80 (9.35%) M(SD) or % (n)	Profile 2: Intermediate WB n = 320 (37.38%) M(SD) or % (n)	Profile 3: Higher WB n = 456 (53.27%) M(SD) or % (n)	Khi^2^ (2) or F(2, 853)	Comparisons (Profile 1 = 1 / Profile 2 = 2 /Profile 3 = 3)
WB					
CASP-12					
Life satisfaction	17.84 (2.93)	25.28 (2.44)	31.73 (2.45)		
Self-perceived health	6.15 (1.73)	6.89 (1.41)	8.06 (1.08)		
	1.79 (0.82)	2.40 (0.82)	3.17 (0.87)		
Chronic illness	0.73 (0.44)	0.68 (0.46)	0.49 (0.5)	17.71***	1 = 2 > 3***
Cognitive abilities					
Verbal fluency [0–100]	14.78 (6,08)	17.38 (6.32)	20.01 (7.57)	26.02***	1 < 2 < 3**
Numeracy score [0–5]	3.17 (1.72)	3.97 (1.37)	4.21 (1.23)	20.74***	1 < 2 < 3**
Learning a list of 10 words	4.21 (1.92)	4.98 (1.68)	5.45 (1.56)	22.13***	1 < 2 < 3***
Delayed recall of a list of 10 words	2.81 (2.26)	3.51 (2.05)	4.07 (2.01)	15.99***	1 < 2 < 3**
Physical health					
Grip strength	22.35 (1.21)	27.07 (0.60)	32.76 (0.51)	46.87***	1 < 2 < 3***
Activity limitation (strongly limited %)	82.5% (n = 66)	64.38% (n = 206)	35.3% (n = 161)	99.52***	1 > 2 > 3**
Engagement with life (YES%)					
Social activities	66.25% (n = 53)	83.44% (n = 267)	91.7% (n = 418)	40.31***	1 < 2 < 3**
Support given	21.71% (n = 17)	32.81% (n = 105)	56.2% (n = 256)	20.35***	1 = 2 < 3***
Support received	39.25% (n = 31)	25.94% (n = 83)	21.25% (n = 97)	42.80***	1 > 2 > 3***
Personality [0–5]					
Openness	3.26 (0.92)	3.45 (0.95)	3.75 (0.91)	15.73***	1 = 2 < 3***
Conscientiousness	4.22 (0.75)	4.37 (0.74)	4.50 (0.68)	6.86**	1 = 2 < 3**
Extraversion	3.27 (0.93)	3.41 (0.86)	3.53 (0.85)	3.87**	1 = 2; 2 = 3; 1 < 3*
Agreeableness	3.80 (0.99)	3.82 (0.79)	3.98 (0.76)	4.48**	1 = 2; 1 = 3; 2 < 3*
Neuroticism	3.77 (1.15)	3.25 (1.03)	2.74 (1.02)	45.58***	1 > 2 = 3***
Sociodemographic variables					
Age	80.11 (7.33)	76.58 (1.66)	72.69 (6.23)	44.92***	1 > 2 > 3**
Gender (female %)	68.75% (n = 55)	62.5% (n = 200)	50.2% (n = 229)	16.89***	1 > 2 > 3***
Marital status (living with spouse/partner %)	36.25% (n = 29)	52.19% (n = 167)	72.6% (n = 331)	56.97***	1 < 2 < 3***
Level of education	2.49 (1.75)	2.25 (1.66)	3.19 (1.71)	27.52***	1 = 2 < 3**
Household income	29,436.53	29,423.04	39,054.37	25.52***	1 = 2 < 3***

^*^p < .05; **p < .01; ***p < .001

#### Components of SA

*Chronic disease*—A one-way ANOVA showed a significant group effect on the number of chronic illnesses (F (2, 853) = 17,711; *p* < 0.001; R^2^ = 0.04). Post hoc analysis indicated that the number of chronic illnesses was significantly higher in Profile 1 than in Profile 3 (*p* < 0.001), and in Profile 2 than in Profile 3 (*p* < 0.001). There were no significant differences between Profiles 1 and 2.

*Cognitive capacity*—A one-way ANOVA showed a significant group effect on verbal fluency (F(2, 853) = 26.021; *p* < 0.001; R^2^ = 0.06). Post hoc analysis indicated that scores in verbal fluency were significantly higher in Profile 2 than 1 (*p* = 0.009), in Profile 3 than 1 (*p* < 0.001) and in Profile 3 than 2 (*p* < 0.001). The results also showed a significant group effect on numeracy scores (F(2, 853) = 20.749; *p* < 0.001; R^2^ = 0.04). In fact, post hoc analysis showed a higher score in Profile 2 than for Profile 1 (*p* < 0.001), higher scores in Profile 3 than in profile 1 (*p* < 0.001), and higher scores in Profile 3 than in Profile 2 (*p* = 0.042). There was a significant group effect on free recall (F(2, 853) = 22.313; *p* < 0.001; R^2^ = 0.05). Post hoc showed that the free recall score was lower in Profile 1 than in Profile 2 (*p* < 0.001) or Profile 3 (*p* < 0.001). The score was also lower in Profile 2 than in Profile 3 (*p* < 0.001). For delayed recall, the ANOVA showed a significant group effect (F(2, 853) = 15,999; *p* < 0.001; R^2^ = 0.04). Indeed, scores were higher in Profile 2 that in Profile 1 (*p* < 0.001) and in Profile 3 than in Profile 1 (*p* < 0.001). A significant difference between Profiles 2 and 3 was observed, with higher scores in Profile 3 (*p* < 0.001).

*Physical functional capacity—*ANOVA showed a significant group effect on grip strength (F (2, 853) = 46.872; *p* < 0.001; R^2^ = 0.10), indicating that all pairwise comparisons were significant, with a higher score in Profiles 2 and 3 than in Profile 1 (*p* < 0.001; *p* < 0.001), and with a higher score in Profile 3 than in Profile 2 (*p* < 0.001). For activity limitation, the Chi^2^ test showed a significant difference between all profiles (χ^2^(2) = 99.52; *p* < 0.001), with higher activity limitation in Profile 1 than Profile 2 (χ^2^(1) = 9.66; p = 0.002) and Profile 3 (χ^2^(1) = 62.08; *p* < 0.001). There was also a significant difference between Profiles 2 and 3, with higher activity limitation in Profile 2 than in Profile 3 (χ^2^(1) = 9.66; *p* < 0.001).

*Engagement with life*—The Chi^2^ test showed a significant difference between groups in social activity (χ^2^(2) = 40.31; *p* < 0.001). People in Profile 1 had less social activities than Profile 2 (χ^2^(1) = 11.82; *p* < 0.001) or Profile 3 (χ^2^(1) = 41.26; *p* < 0.001). The results showed that people in Profile 2 had less social activities than people in Profile 3 (χ^2^(1) = 12.30;*p* < 0.001). Concerning support given, we observed a significant effect (χ^2^(2) = 20.35; p < 0.001). In fact, people in Profile 1 gave less support than people in Profile 3 (χ^2^(1) = 9.51; *p* = 0.002). In addition, Profile 2 gave less support than Profile 3 (χ^2^(1) = 14.91; *p* < 0.001), but there was no significant difference between Profiles 1 and 2. There was a significant difference between profiles in terms of receiving support (χ^2^(2) = 42.80; *p* < 0.001). Indeed, people in Profile 2 received less support than Profile 1(χ^2^(1) = 15; *p* < 0.001) or than Profile 3 (χ^2^(1) = 41.32; *p* < 0.001). The results also showed that people in Profile 2 received more support than people in Profile 3 (χ^2^(1) = 11.96; *p* < 0.001).

#### Personality

ANOVA showed a significant group effect on openness (*F*(2, 853) = 15.73; *p* < 0.001; R^2^ = 0.03). Post hoc showed that Profile 3 displayed higher openness (*p* < 0.001; *p* < 0.001) scores than people in Profile 1 and Profile 2, but no significant difference was observed between Profiles 1 and 2. A significant group effect on conscientiousness was observed (F(2, 853) = 6.865; *p* = 0.001; R^2^ = 0.01), indicating that people in Profile 3 had higher conscientiousness scores than people in Profile 1 (*p* = 0.011) or than people in Profile 2 (*p* = 0.027). There was no significant difference between Profiles 1 and 2. Concerning extraversion scores, ANOVA showed a significant group effect (F(2, 853) = 3.875; *p* = 0.021; R^2^ = 0.009) and post hoc showed a significant difference only between Profiles 1 and 3, with a higher score in Profile 3 (*p* = 0.038). Likewise, the one-way ANOVA showed a significant group effect on agreeableness (F(2, 853) = 4.476; *p* = 0.12; R^2^ = 0.01). There was a significant difference between Profile 2 and Profile 3 (*p* = 0.017), with a higher score in people in Profile 3. However, there was no significant difference between Profiles 1 and 2, or between Profiles 1 and 3. Finally, ANOVA showed a significant group effect on neuroticism (F(2, 853) = 45.058; *p* < 0.001; R^2^ = 0.09), indicating that people in Profile 1 had a significantly higher score than people in Profile 2 (*p* < 0.001) or than people in Profile 3 (*p* < 0.001). There was no significant difference between Profiles 2 and 3.

#### Sociodemographic variables

ANOVA showed a significant group effect on age (F(2, 781) = 44.925; *p* < 0.001; R^2^ = 0.10). Post hoc showed that there was a significant age difference between all profiles (all pairwise comparisons were significant at *p* < 0.001). People in Profile 3 were younger than people in Profile 1 or 2. Gender was significantly different between Profiles 1 and 2, and between Profiles 2 and 3 (χ^2^(2) = 16.89; *p* < 0.001). There were more women in Profile 1 than in profile 3 (χ^2^(1) = 9.38; p = 0.002), in Profile 2 than in Profile 3 (χ^2^(1) = 11.47; *p* < 0.001). The results showed a significant difference in marital status between all profiles (χ^2^(2) = 56.97; *p* < 0.001). People in Profiles 2 and 3 were more likely to live with a spouse/partner than in Profile 1 (χ^2^(1) = 6.51; *p* = 0.01; (χ^2^(1) = 40.75; *p* < 0.001). People in Profile 3 were more likely to live with a spouse/partner than those in Profile 2 (χ^2^(1) = 34.04; p < 0.001). There was also a significant effect of the level of education (F(2, 773) = 27.517; *p* < 0.001; R^2^ = 0.07). Post hoc analyses showed no difference between Profiles 1 and 2, but a higher level in Profile 3 than in Profile 1 (p = 0.01), and between Profiles 2 and 3, with a higher level in Profile 3 (*p* < 0.001). Finally, a significant group effect on household income was found (F(2, 853) = 25.519; *p* < 0.001; R^2^ = 0.05), with higher household income for people in Profile 3 than for those in Profile 1 (*p* < 0.001). There was also a significant difference between Profiles 2 and 3 (*p* < 0.001): household income was higher in Profile 3 than in Profile 2. There was no significant difference between Profiles 1 and 2.

## Discussion

The first aim of this study was to identify different profiles based on older adults’ WB. To our knowledge, this is the first study that has objectified WB profiles in older people using these determinants. The results allowed us to identify three profiles of WB. 53.27% of the sample were included in Profile 3 (higher WB). People who fit this profile could be considered to be healthy aging. However, it is important to point out that people belonging to Profile 3 were younger, had higher incomes, a higher level of education, and lived mainly with their spouse/partner. The results showed better scores in terms of physical activity, cognitive functioning, social resources and individuals from this group were more open, conscientious, extroverted and pleasant. These results are therefore consistent with the impact of healthy aging on WB [36]. The results also highlighted that the majority of older adults in our cohort had high levels of WB, as only 9% (n = 80) reported lower levels of WB. These findings are consistent with standard medical criteria (good health equals WB) and with the Rowe and Kahn’ SA model. In contrast to Profile 3, Profile 1 (lower WB) has lower scores in terms of "SA" according to the variables of the Rowe and Kahn model. The same is true in terms of personality and socio-demographic variables. This raises questions about the injunctions concerning healthy aging that older adults integrate into their lives. Indeed, while the subjectivity of the individual was taken into account here with the WB profiles, the results tend to show that people generally follow Rowe and Kahn's SA model. One could wonder whether the normative criteria of SA have been integrated by older people as absolute criteria for successful aging. Moreover, the absence of cut-off points for WB assessments may also be a limitation of our study, as it raises the question of when we can distinguish high from low WB. Finally, while the concept is useful for understanding how individuals can increase their chances of healthy aging, it is questionable whether individuals adopt healthy behaviors because they receive this message from prevention systems in particular. It may be that the focus on creating environments that enhance the lives of aging people sensitizes individuals to the kinds of behaviors they should adopt to increase the likelihood of successful aging [37].

Our results also suggest that there are different ways to age well, with an intermediate level of WB (37.38%, n = 320). It's also interesting to note that the intermediate profile (profile 2) can be either close to the "lower WB" profile in terms of openness, conscientiousness, agreeableness or to the "higher WB" profile in terms of extraversion. But the three profiles do not have the same level of neuroticism. This means that even people belonging to the intermediate profile are less open or aware, they are not neurotic. The results showed that the scores do not differ in the other two profiles in terms of neuroticism, despite a difference in age, which means that the intermediate WB profile is again sometimes close to the high WB profile and sometimes close to the low WB profile. The intermediate profile is therefore an interesting one on which we could act with interventions to avoid the decline of positive traits in order to prevent an eventual transition to a lower level of WB.

These results are therefore interesting from a clinical point of view, as they show the importance of taking personality traits, into account when working with non-institutionalized and independent older adults, particularly neuroticism, when looking at a WB. From a theoretical point of view, PWB and SWB seem to be different concepts, emphasizing, on the one hand, the achievement of goals to improve quality of life and, on the other, the subjective interpretation of WB. Finally, these results also underline the importance of considering people belonging to Profile 2, which could be considered as a specific profile, especially in terms of personality traits. These results also showed the importance of adapting the support offered to older people according to their health status and/or individual characteristics and we note that it is important to add psychological variables (such as personality) to account for the subjectivity of the individual.

## Limitations and perspectives

This study is important to help understanding the factors influencing WB in older adults. However, we must acknowledge some limitations. First, the choice of our measures could have influenced the findings, particularly concerning the measures of engagement with life. In this study, we used social activities, and support given/received, but Rowe and Kahn’s model mentions two forms of engagement with life: interpersonal relations and productive activity. Thus, we could have used more appropriate measures for these two forms of engagement, but the SHARE database did not allow us to do so. Second, although the sample closely matched the male/female distribution of the national population, the participants all lived at home which may not be representative of the population as a whole. In particular, socioeconomic status (income) may create conditions that favor healthy aging, such as managing stress or coping with negative emotions, that could explain the small proportion of individuals including in Profile 1 [38, 39]. Future studies could also investigate the WB of people living in residential homes or in residential care for older adults to observe dependent older adults, with less standardized criteria than the actual study. Indeed, all our participants lived at home and were autonomous in their daily life, which may have influenced our results, particularly regarding cognitive and physical abilities, but also the social environment. We also need to be cautious about measuring marital status and its effect on the well-being of older people. While people who are married or living with someone are less likely to feel lonely, loneliness is not synonymous with social isolation [40, 41]. Finally, we looked at household income, a factor that can be influenced by whether or not individuals live with someone in their household. This is a limitation, as it is strongly conditioned by whether or not both people work, for example.

The originality of our study was to treat several variables together and not separately. However, it seems important to emphasize that not all variables associated with healthy aging were considered here. In particular, it might be interesting in the future to look at the impact of subjective age on the WB of older people, in order to promote a subjective approach to aging and understand certain health behaviours. In addition, the adaptation process and mental flexibility are interesting variables explore, because they would enable us to understand the adaptation processes put in place by the individual to cope with the events encountered during aging, and the health behaviors that may result.

Finally, it is important to note that the data in this study was collected over a specific period in 2017 and does not represent changes over a person's lifetime. However, SHARE is a multi-waves study, thus the next studies could focus on the life trajectories of individuals in order to understand inter-individual differences that may change over the life course and aging, and the determinants of WB.

## Conclusion

This study contributes to the debate surrounding the notion of SA and confirms that SA occupies an important place in the context of healthy aging, although other factors must also be considered. Indeed, personality traits can contribute to positive functioning in terms of health, cognition, social relationships, and WB across the lifespan [18], and thus enables individuals to engage in healthy behaviors that promote health outcomes and thus WB. Overall, we observed that the majority of non-institutionalized and independent older people in our study are aging in good health, which raises questions about the adoption of certain health behaviors conveyed by the "high WB equals good health" message. Also, the results highlighted the importance of acting upstream and adapting prevention policies for healthy aging on the person's own terms, so that everyone can live a long and pleasant life and limit disparities in old age.

## Data Availability

The experimental data and results supporting the conclusions of this study are available in SHARE with the identifier 10.6103/SHARE.easy.800 at the following https://share-eric.eu/data/.
